# Breaking Barriers: The Life and Legacy of Gerty Cori in Biochemical Research

**DOI:** 10.7759/cureus.69409

**Published:** 2024-09-14

**Authors:** Sara Tahir, Sana Tahir, Latha Ganti

**Affiliations:** 1 Physiology and Kinesiology, University of Florida, Gainesville, USA; 2 Medicine, Orlando College of Osteopathic Medicine, Winter Garden, USA; 3 Emergency Medicine and Neurology, University of Central Florida, Orlando, USA; 4 Research, Orlando College of Osteopathic Medicine, Winter Garden, USA; 5 Medical Science, The Warren Alpert Medical School of Brown University, Providence, USA

**Keywords:** anaerobic glycolysis, carbohydrate metabolism, cori cycle, cori ester, "historical vignette", historical vignette

## Abstract

Gerty Theresa Cori, a remarkable and pioneering biochemist, became the first woman to receive the Nobel Prize in Physiology or Medicine in 1947 for her groundbreaking research in carbohydrate metabolism. Her work, in collaboration with her husband Carl Ferdinand Cori, revolutionized the scientific understanding of carbohydrate metabolism and had a profound impact on medicine and human health. This paper offers a historical vignette of Gerty Cori's life, tracing her journey from her early years in Prague to her pivotal role in transforming biochemistry. It highlights her immense dedication to scientific research, overcoming significant gender-based challenges, and establishing a legacy that continues to inspire. Gerty Cori's contributions to science not only advanced our knowledge of metabolic processes but also paved the way for future generations of researchers, particularly women in science, technology, engineering, and mathematics.

## Introduction and background

Gerty Theresa Cori, born on August 15, 1896, in Prague, Czechoslovakia, was the eldest of three sisters in an upper-middle-class Jewish family. Her father, Otto Radnitz, was a chemist and businessman who managed sugar refineries. Her mother, Martha Neustradt, had a brother who was a professor of pediatrics at the University of Prague, inspiring Gerty's passion for the sciences and influencing her career choice. Initially homeschooled until the age of 10, Gerty later attended a Lyceum for young girls in 1906, from which she graduated in 1912. Despite her graduation, she found herself unprepared for medical school, prompting her to undertake a concentrated two-year study at Tetschen Realgymnasium at the age of 18. After successfully passing her final examinations, she applied to the German University of Prague's medical school. It was there that she met Carl Ferdinand Cori, a fellow medical student who would become both her husband and research partner. They married in August 1920, the same year Gerty earned her doctorate in medicine. Together, Gerty and Carl embarked on a remarkable scientific collaboration, producing their first joint publication in 1920, which marked the beginning of their profound research contributions to science [1-7].

## Review

Early research and the decision to emigrate

After graduating from medical school, Gerty Cori worked as a research assistant under Professor W. Knoepfelmacher at the Karolinen Children’s Hospital in Vienna. During her two years there, she published several research papers on hematological dyscrasias and focused on studying temperature regulation in patients with congenital myxedema. She also conducted multiple experiments on rabbits that had undergone thyroidectomies. Meanwhile, Carl worked as an assistant in pharmacology at the University of Graz for a year. During this time, he encountered antisemitic sentiment, revealed in the form of challenges in securing academic positions at universities. These experiences motivated both Gerty and Carl to emigrate to the United States in 1922 [1-7].

The Coris pioneering transition to carbohydrate metabolism research

In 1922, Carl Cori emigrated to Buffalo, New York, where he joined the New York Institute for the Study of Malignant Disease. Six months later, his wife, Gerty, joined him and assumed the role of assistant pathologist at the same institution. Initially, their collaborative research as a couple faced resistance, but over time, their colleagues accepted their partnership, enabling them to work together harmoniously. In 1923, Gerty Cori published her first work in the United States, The Influence of Thyroid Extract and Thyroxine on the Rate of Multiplication of Paramecia. While in New York, the Coris began to shift their focus to carbohydrate metabolism, embarking on a three-decade research journey. Their work included numerous publications on liver metabolism, the role of galactose in the presence of glucose, and the comparison of blood sugar levels during insulin action [1-7].

Facing gender discrimination: Gerty Cori's resilience in academia

**Figure 1:**
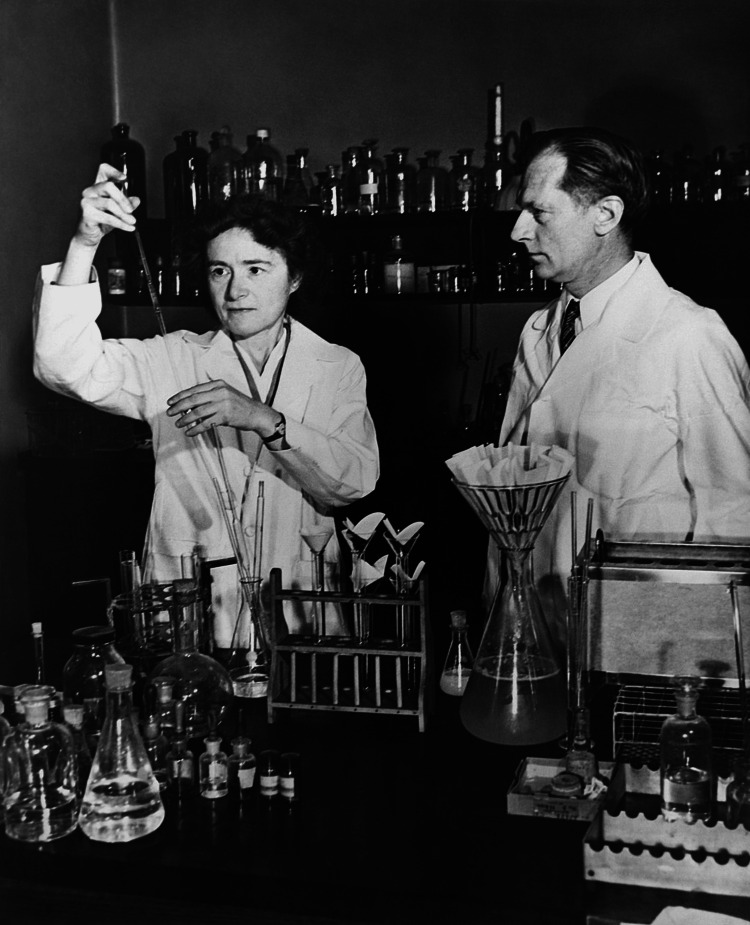
Gerty Cori and her husband Carl Cori in the laboratory at Washington University School of Medicine, St. Louis, 1947 Image Source: Jewish Women's Archive [3]

In 1931, Carl Cori became the chairman of the Department of Pharmacology at Washington University School of Medicine, where Gerty Cori was also offered a research position. Due to the university’s policy allowing only one family member to hold a faculty position, Gerty was hired as a research fellow instead. Despite working in the same laboratory and performing equivalent duties, Gerty was paid only 10 percent of her husband's salary. This stark salary discrimination highlights one of the significant challenges Gerty Cori had to endure and overcome as a female in the field. Her resilience and determination to continue and excel in a male-dominated field not only showcase her excellence but also affirm her invaluable contributions as a woman in medicine [3,6]. Figure 1 below depicts Gerty and Carl Cori working in the laboratory while at the Washington University School of Medicine.

The Cori cycle: revolutionizing carbohydrate metabolism

Over the next few decades, the Coris published numerous research papers while continuing their study of carbohydrate metabolism at Washington University. Before their groundbreaking work, the scientific understanding of glycogen conversion to glucose was largely attributed to hydrolysis mechanisms. While it was generally known that ineffective carbohydrate metabolism could lead to diabetes mellitus, the underlying biochemical processes were not well understood. In the 1930s, the Coris made a pivotal discovery now known as the "Cori Cycle." This cycle describes the biochemical mechanism of carbohydrate metabolism, where glucose is broken down into pyruvate during anaerobic glycolysis and then converted into lactate. The lactate is transported through the bloodstream to the liver, where it is converted back into pyruvate. Through gluconeogenesis, this pyruvate is transformed into glucose, which is then transported back to the muscles, completing the cycle. This discovery was transformative, revolutionizing the scientific community’s understanding of carbohydrate metabolism [1,2,5,6,7].

Pioneering discoveries in glycogen metabolism: the Cori ester and key enzymes

In 1936, the Coris expanded their research to include hexose monophosphate in glycogenolysis and made a pivotal discovery by isolating glucose-1-phosphate, commonly known as the "Cori ester." They linked glucose-1-phosphate to the action of glycogen phosphorylase, an enzyme responsible for catalyzing the degradation and synthesis of polysaccharides. Additionally, the Coris discovered phosphoglucomutase, an enzyme that facilitates the conversion between glucose-1-phosphate and glucose-6-phosphate. These breakthroughs illuminated the synthesis pathways of glycolysis and glycogenolysis, emphasizing the profound impact of the Coris' contributions to science [1,2,4].

Gerty Cori: breaking barriers in science and academia

In 1936, Gerty Cori gave birth to her only son, Thomas Cori. Despite the demands of motherhood, she remained deeply committed to her research, showcasing her unwavering dedication and passion for science. In the 1940s, both Gerty and Carl transitioned to the Department of Biological Chemistry, where Gerty was appointed as an associate professor of Research Biological Chemistry and Pharmacology in 1943. She was later promoted to full professor in July 1947. That same year, Gerty and Carl were awarded the Nobel Prize in Physiology or Medicine. Gerty was the first woman to receive this prestigious award, solidifying her legacy as a pioneering and influential figure within both the scientific community and beyond [2-7].

The lasting legacy of the Coris: mentorship and final contributions

Many scientists were eager to collaborate with the Coris after the publication of their groundbreaking research. Among the numerous scientists mentored by the Coris, six went on to receive Nobel Prizes, underscoring the profound influence and lasting legacy the Coris had on the scientific community. In the final decade of her life, Gerty Cori continued her pioneering work, focusing on the study of enzyme defects in inherited glycogen storage diseases in pediatric patients. Sadly, on October 26, 1957, Gerty Cori passed away after a decade-long battle with myelofibrosis [3,4,6,7].

## Conclusions

Gerty Cori's legacy stands as a powerful testament to her extraordinary contributions to biochemistry, achieved in collaboration with her husband, Carl Cori. As the first American woman to receive the Nobel Prize in Physiology or Medicine, she earned this recognition for her groundbreaking work in carbohydrate metabolism, including the discovery of the "Cori cycle" and the "Cori ester." These discoveries not only advanced scientific understanding but also laid the foundation for future generations of researchers. Her perseverance in the face of gender discrimination and her unwavering commitment to scientific excellence continue to inspire and shape the scientific community. Gerty Cori's life and work truly demonstrate the profound impact that one determined individual can have on the advancement of science and human knowledge.
